# ﻿The pseudoscorpions of the Caucasian *Sphagnum* bogs: part I. Description of Neobisium (Neobisium) adjaricum sp. nov. and redescription of the holotype of N. (N.) vilcekii Krumpál, 1983 (Arachnida, Pseudoscorpiones, Neobisiidae)

**DOI:** 10.3897/zookeys.1100.81910

**Published:** 2022-05-16

**Authors:** Vasiliy B. Kolesnikov, Jana Christophoryová, Andrey A. Przhiboro, Ilya S. Turbanov

**Affiliations:** 1 Federal Public Budgetary Scientific Institution All-Russian Research Institute of Protection of Plants, VNIISS, Voronezh Province, 396030, Russia Federal Public Budgetary Scientific Institution All-Russian Research Institute of Protection of Plants, VNIISS Voronezh Russia; 2 Tyumen State University, 6 Volodarskogo Str., Tyumen, 625003, Russia Tyumen State University Tyumen Russia; 3 Department of Zoology, Faculty of Natural Sciences, Comenius University, Mlynská dolina, Ilkovičova 6, SK–842 15 Bratislava, Slovakia Comenius University Bratislava Slovakia; 4 Zoological Institute, Russian Academy of Sciences, Universitetskaya nab. 1, Saint Petersburg, 199034, Russia Zoological Institute, Russian Academy of Sciences Saint Petersburg Russia; 5 I.D. Papanin Institute of Biology of Inland Waters, Russian Academy of Sciences, Borok, Yaroslavl Province, 152742, Russia I.D. Papanin Institute of Biology of Inland Waters, Russian Academy of Sciences Borok Russia; 6 Cherepovets State University, Vologda Province, Cherepovets, 162600, Russia Cherepovets State University Cherepovets Russia

**Keywords:** Bryobiont, Georgia, mire, new species, peat bog, Russia, taxonomy, tyrphophile

## Abstract

A new species of pseudoscorpions, Neobisium (Neobisium) adjaricum**sp. nov.**, is described and diagnosed. It was collected in the *Sphagnum* habitats of Ispani lowland mires in Transcaucasia (Republic of Adjara, Georgia). The habitat of N. (N.) adjaricum**sp. nov.** is described. The holotype of N. (N.) vilcekii Krumpál, 1983, a species most similar morphologically to N. (N.) adjaricum**sp. nov**., known from the North Caucasus (Republic of North Ossetia–Alania, Russia), is redescribed. Diagnostic characters of the relative Caucasian species of the subgenus Neobisium Chamberlin, 1930 are analysed.

## ﻿Introduction

Pseudoscorpions are well known to inhabit peat bogs, but the fauna of pseudoscorpions in bogs is peculiar and poor. It includes both specialised tyrphophilous bryobionts (i.e., species associated mostly with moss habitats in mires) and eurytopic species (Turbanov et al. 2017). The pseudoscorpions of the Caucasus are relatively well studied, but they were not investigated as a component of the biodiversity of *Sphagnum* bogs in this region.

The Caucasus is one of the biodiversity hotspots (Myers et al. 2000), with a high variety of landscapes and habitats. The mires of the Caucasus, including *Sphagnum* bogs, are relatively rare, isolated, mostly small-sized ecosystems with specific conditions (Botch and Masing 1979, 1983). Peat bogs in the Caucasus are peculiar also because they are situated near the southernmost limit of the occurrence of this habitat type in the Palaearctic Region. Most of Caucasian bogs are located at an altitude between 600 and 3,400 m a.s.l. In contrast, the *Sphagnum* bogs of western Georgia (Transcaucasia) strongly differ from other Caucasian bogs in many important features (Krebs et al. 2017; Sirin et al. 2017). They are situated in the Kolkheti (= Colchis) Lowland, near the Black Sea coast, at an altitude of less than 10 m a.s.l. These bogs are unique as they are the only *Sphagnum* bogs situated in the subtropical climatic zone, representing a separate Kolkheti (= Colchis) mire region (Botch and Masing 1983; Tanneberger et al. 2021). Both montane Caucasian and lowland Transcaucasian bogs are considered to contain relict biotic components and to serve as postglacial refugia for species of boreal origin, primarily plants (Denk et al. 2001; Kaffke 2008; Doroshina and Nikolajev 2018). Hitherto, terrestrial arthropods remain almost unstudied in the peat bogs of the Caucasus and, consequently, any contribution to their knowledge is important.

**Figure 1.**
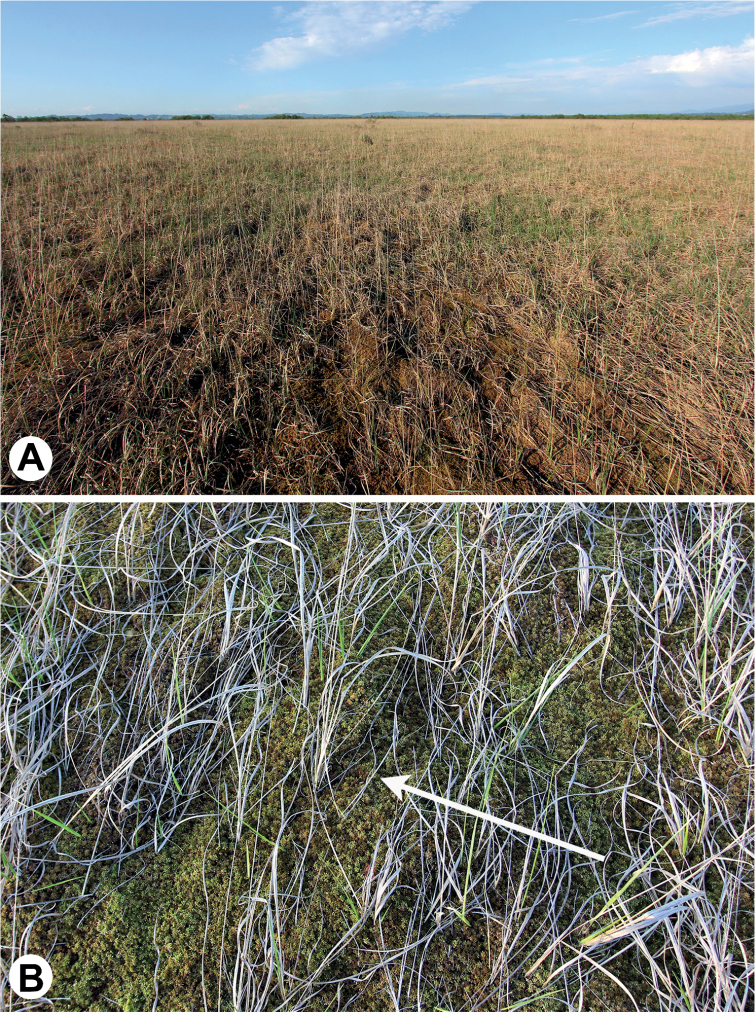
The habitat of Neobisium (Neobisium) adjaricum sp. nov.: Ispani 2 bog near the town of Kobuleti (Republic of Adjara, Georgia) **A** general view of site 3 (10 May 2019) **B***Sphagnum* carpet, with exact location where the holotype was collected (sample I-11) indicated by an arrow (10 May 2019). Photographs by AP.

**Figure 2.**
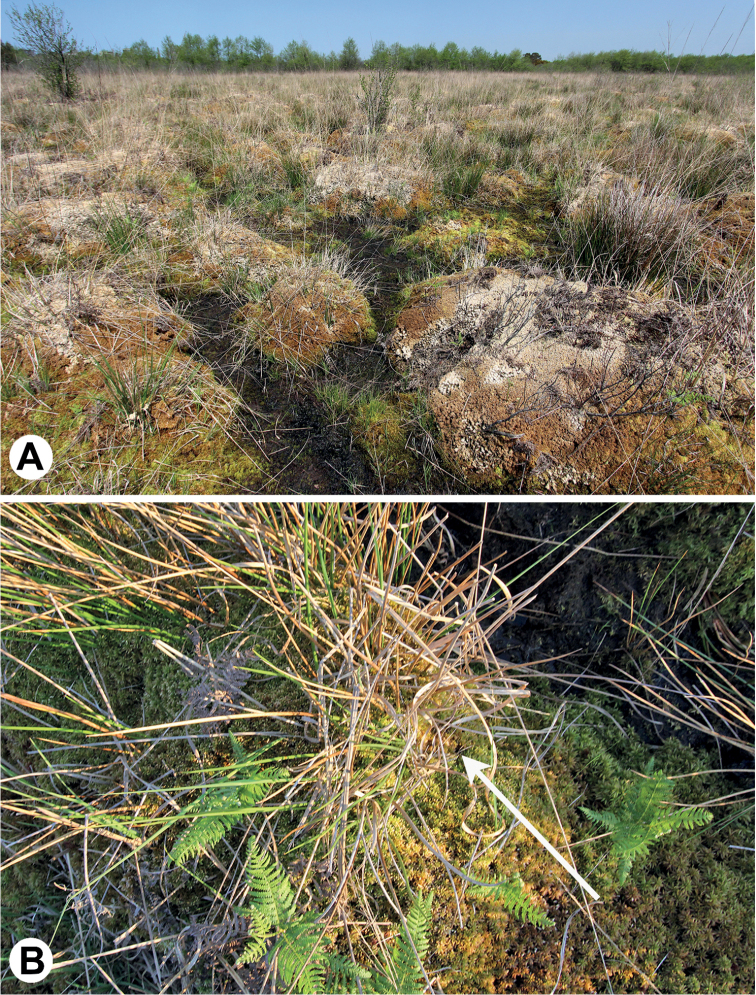
The habitat of Neobisium (Neobisium) adjaricum sp. nov.: Ispani 1 bog near the town of Kobuleti (Republic of Adjara, Georgia) **A** general view of site 1 (30 April 2019) **B***Sphagnum* hummock, with exact location where two paratypes were collected (sample I-18) indicated by an arrow (11 May 2019). Photographs by AP.

The present paper is first contribution to the knowledge of pseudoscorpions inhabiting the mires of the Caucasus, based on the original material collected in the scope of ecological and faunal studies of ten bogs. A species new to science described below, Neobisium (Neobisium) adjaricum sp. nov., has been collected from Ispani 1 and Ispani 2 (Figs 1A, 2A) bogs situated in the Kolkheti Lowland of Georgia. In addition, we re-examined and redescribed holotype of N. (N.) vilcekii Krumpál, 1983, species most similar morphologically to N. (N.) adjaricum sp nov., described from the North Caucasus (near Karmadon in the Republic of North Ossetia–Alania, Russia; Krumpál 1983). Nassirkhani and Snegovaya (2021) recently redescribed N. (N.) vilcekii based on the new material collected near Mozdok, Republic of North Ossetia–Alania, ca. 100 km far away from the type locality. However, redescription of the holotype highlights important diagnostic characters and demonstrates an intraspecific variability of this species as compared with the redescription provided by Nassirkhani and Snegovaya (2021).

The subgenus Neobisium Chamberlin, 1930 currently contains 119 recent and two fossil species distributed around the world (World Pseudoscorpiones Catalog 2022), of which 19 are found in the Caucasus: Neobisium (N.) alticola Beier, 1973 (Azerbaijan), N. (N.) anatolicum Beier, 1949 (Republic of North Ossetia–Alania, Russia; Armenia; Azerbaijan; Georgia), N. (N.) artaxerxesi Nassirkhani, Snegovaya & Chumachenko, 2018 (Republic of Adygea, Russia), N. (N.) carcinoides (Hermann, 1804) (North Caucasus, Russia; Georgia), N. (N.) catherineae Nassirkhani, Zaragoza, Snegovaya & Chumachenko, 2020 (Krasnodar Territory, Russia), N. (N.) crassifemoratum (Beier, 1928) (Azerbaijan), N. (N.) erythrodactylum (L. Koch, 1873) (Armenia; Azerbaijan; Georgia), N. (N.) fuscimanum (C.L. Koch, 1843) (Georgia), N. (N.) golovatchi Schawaller, 1983 (Krasnodar Territory, Russia), N. (N.) granulatum Beier, 1937 (North Caucasus including its southern macroslope within the Krasnodar Territory, Russia; Georgia; Azerbaijan), N. (N.) kamenskyi Nassirkhani, Zaragoza, Snegovaya & Chumachenko, 2020 (Krasnodar Territory, Russia), N. (N.) kobachidzei Beier, 1962 (North Caucasus, Russia; Georgia; Azerbaijan), N. (N.) kovalevskayae Nassirkhani, Snegovaya & Chumachenko, 2019 (Krasnodar Territory, Russia), N. (N.) labinskyi Beier, 1937 (Stavropol Territory, Russia; Azerbaijan; Georgia), N. (N.) macrodactylum (Daday, 1888) (Azerbaijan), N. (N.) speleophilum Krumpál, 1986 (Krasnodar Territory, Russia), N. (N.) sylvaticum (C.L. Koch, 1835) (Republic of North Ossetia–Alania, Russia; Georgia; Azerbaijan), N. (N.) validum (L. Koch, 1873) (Armenia; Azerbaijan), and N. (N.) vilcekii Krumpál, 1983 (Republic of North Ossetia–Alania, Russia) (Dashdamirov and Schawaller 1992; Nassirkhani et al. 2020; World Pseudoscorpiones Catalog 2022).

Dashdamirov and Schawaller (1992) and Nassirkhani et al. (2020) indicated that the distribution of the subgenus Neobisium within the Caucasus was poorly known and this taxon is likely to be more diverse in the area than indicated by the published records. The results of our study confirm this and contribute to the diversity of pseudoscorpions in the Caucasus.

## ﻿Study bogs

The *Sphagnum* bogs Ispani 1 and Ispani 2 (Figs 1A, 2A) are situated in the Kolcheti Lowland, near the town of Kobuleti in the Republic of Adjara, Georgia, western Transcaucasia, ca. 1 km of the Black Sea coast. This area is characterised by a high mean annual temperature (13–14 °C), it is almost without frosts in winter, with very high levels of mean annual precipitation (ca. 1,500–2,500 mm), with ca. 50% of the precipitation falling from May to October, and with high air humidity (Kaffke 2008; de Klerk et al. 2009).

The bogs have an area of ca. 4.6 and 2.5 km^2^, respectively (Krebs et al. 2017). The natural conditions, vegetation and environmental history of Ispani 1 and Ispani 2 are relatively well documented (Kaffke 2008; de Klerk et al. 2009; Krebs et al. 2009; and references therein). These bogs are ombrotrophic (only rain-fed), acidic and oligotrophic, with *Sphagnumpapillosum* Lindb., *S.austinii* Sull., and *S.palustre* L. predominating in the vegetation cover.

The Ispani 1 bog (Fig. 2A) is strongly modified due to human activity during the 20^th^ century (drainage and peat excavation) and is now considered as “degraded”; it has a well-developed relief of hummocks and interspaces (Fig. 2B). The Ispani 2 bog (Fig. 1A) is relatively undisturbed and retains its natural state. It is famous for belonging to a unique mire type, percolation bog, which does not have surficial, but predominantly only vertical water flow through the entire peat body. As a consequence, the surface is nearly flat over most of the bog, without explicit microhabitats (Fig. 1B).

## ﻿Materials and methods

Four specimens of a new species of the subgenus Neobisium (3 ♂ and 1 ♀) were collected from the bogs Ispani 1 and Ispani 2 briefly described above. All specimens were taken from quantitative samples of substrate consisting mostly of *Sphagnum*, taken up to a depth of 20–30 cm from the surface. Samples were washed in sieves (the smallest mesh size 0.25 × 0.25 mm), and then macroinvertebrates were extracted by flotation in a strong solution of NaCl combined with hand-sorting of the coarse fraction (see e.g., Khalin et al. (2022) for further details of flotation). All specimens were kept in 85% ethyl alcohol. Habitat parameters were measured in the water squeezed from *Sphagnum* at the sample point: pH value by a Hanna pHep + pH meter and mineralisation (ppt) by a Hanna DIST 2 conductometer.

For morphological examination using light microscopy, Neobisium (Neobisium) adjaricum sp. nov. was cleaned in pure lactic acid and was temporarily mounted on microscopic slides in glycerol. Some specimens were dissected for a more detailed study of the chelicerae, pedipalps and legs I and IV. The drawings were made under a Biomed 6 microscope (variant 3). The photographs of habitus were taken with a Leica MC170 HD (12MPs) digital microscope camera using the extended focus technology (Helicon Focus 7.7.4). After the study, each specimen including the dissected body parts was returned to the vial containing 85% ethanol.

**Figure 3.**
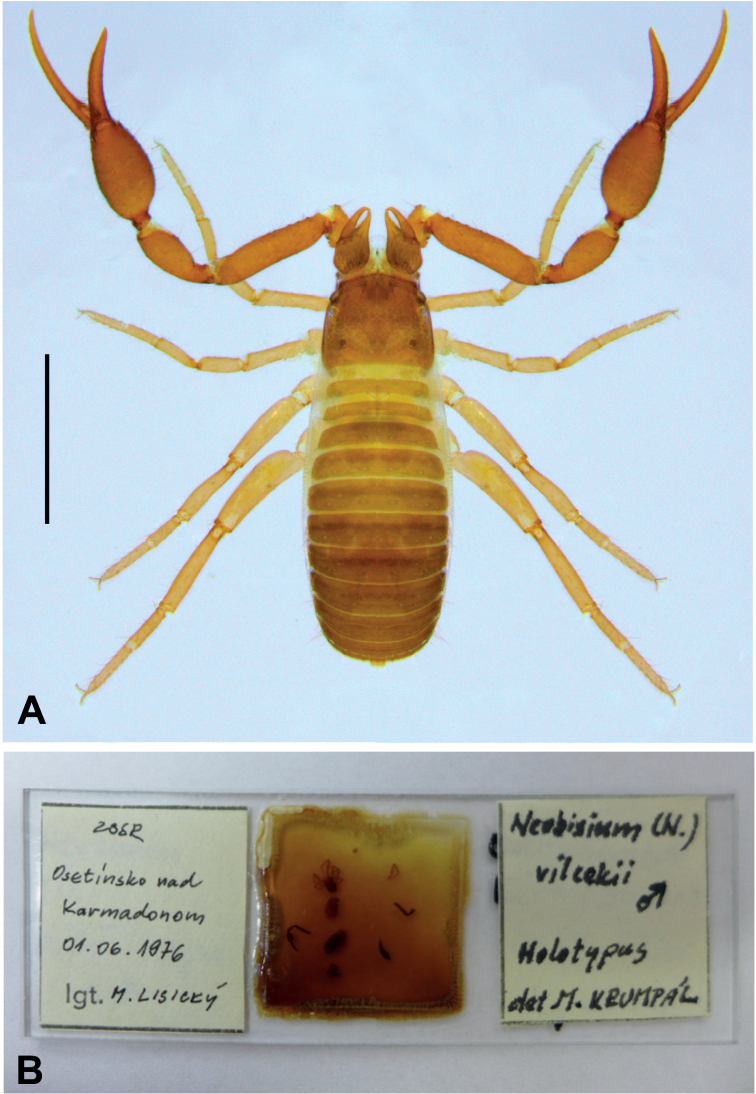
**A** habitus of Neobisium (Neobisium) adjaricum sp. nov., holotype male, dorsal view **B** permanent slide mount with Neobisium (Neobisium) vilcekii Krumpál, 1983, holotype male. Scale bar: 1 mm.

The holotype of N. (N.) vilcekii, deposited in the zoological collection of the Slovak National Museum (**SNM**) in Bratislava, Slovakia, is redescribed. A permanent slide mount containing the dissected type species was studied (Fig. 3B) (pedipalps, chela, chelicera, leg IV, and abdomen separated). The slide bears the following labels: “Neobisium (N.) vilcekii, male, Holotypus, det. M. Krumpál” (right) and “ZSSR, Osetínsko nad Karmadonom, 01.06.1976, lgt. M. Lisický” (left). Morphological and morphometric analyses were performed using a Leica DM1000 compound microscope with an ICC50 Camera Module (LAS EZ application, 1.8.0). Measurements were taken from digital images using the AxioVision 40LE application. Drawings were generated using a Leica DM1000 drawing tube. Image stacks were produced manually, combined using the Zerene Stacker software, and edited with Adobe Photoshop CC.

The type material of N. (N.) adjaricum sp. nov. is deposited in the following collections: Zoological Institute, Russian Academy of Sciences, Saint Petersburg (**ZISP**); Zoological Museum of the Moscow University (**ZMMU**); and Institute of Systematics and Ecology of Animals, the Siberian Branch of the Russian Academy of Sciences, Novosibirsk (**ISEA**).

The measurements were made with an ocular micrometre using the reference points proposed by Chamberlin (1931) and those are given in millimetres (mm). All measurements are presented either as length/width ratios (carapace, chelicera, and pedipalp) or as length/depth ratios (legs). Proportions are indicated by the symbol “×”. The data on paratypes are given in parentheses, after the holotype data.

Morphological terminology follows Chamberlin (1931), with amendments proposed by Harvey (1992), Harvey and Edward (2007) and Judson (2007a; 2017). Inclusion of the pedicel in the measurement data of the chela is indicated by a plus sign (e.g., hand^+^) and its exclusion by a minus sign (e.g., hand–) (Judson 2007b).

### ﻿The abbreviations are as follows

***as, bs, es, gs, is, ls, sbs*** cheliceral setae;

***dat*** dorsal accessory tooth on pedal tarsus IV;

***fa*** retrolateral lyrifissure of fixed chelal finger;

***fb*** dorsoretrolateral lyrifissure of fixed chelal finger;

***fd*** dorsodistal lyrifissure of fixed chelal finger;

***m*** microsetae;

***ma_1_*, *ma_2_*, *ma_3_*** retrolateral lyrifissures of movable chelal finger;

***pc*** coupled sensillum;

**T** tactile seta;

***tis*** teeth under interior seta *is* of chelicera.

#### Trichobothriotaxy:

***b*** basal trichobothrium;

***eb*** external basal trichobothrium;

***esb*** external sub-basal trichobothrium;

***est*** external sub-terminal trichobothrium;

***et*** external terminal trichobothrium;

***ib*** internal basal trichobothrium;

***isb*** internal sub-basal trichobothrium;

***ist*** internal sub-terminal trichobothrium;

***it*** internal terminal trichobothrium;

***sb*** sub-basal trichobothrium;

***st*** sub-terminal trichobothrium;

***t*** terminal trichobothrium.

## ﻿Results

### ﻿Family Neobisiidae Chamberlin, 1930

**Genus *Neobisium* Chamberlin, 1930**

#### Subgenus Neobisium Chamberlin, 1930

##### ﻿ Neobisium (Neobisium) adjaricum sp. nov.

Taxon classificationAnimaliaPseudoscorpionesNeobisiidae

4DC9B50A-46B7-56A2-BA24-98403F8C8405

http://zoobank.org/A66BABF5-698C-4B29-888D-12945CE6FADB

Figs 3A , 4 , 5 , 6

###### Material examined.

***Holotype*.** ♂ (ZISP 1437), **Georgia**, Adjara, near Kobuleti, Ispani 2 bog, 41.86461°N, 41.79763°E, main bog, site 3 (600 m E of W *Alnus* belt), flat *Sphagnum* carpet, sample I-11, 10.V.2019, A. Przhiboro leg.

***Paratypes*.** 1 ♂ (ZMMU TI-65), 1 ♀ (ZISP 1438), **Georgia**, Adjara, near Kobuleti, Ispani 1 bog, 41.85805°N, 41.78750°E, site 1 (150 m E of NW bog edge), dry *Sphagnum* hummocks, sample I-18, 11.V.2019, A. Przhiboro leg.; 1 ♂ (ISEA Ps. 001.0037), the same locality, habitat, and collector, sample I-37, 10.X.2019.

###### Diagnosis.

Carapace with moderately long and apically rounded epistome; two pairs of eyes present, lenses of posterior eyes with low convexity; movable cheliceral finger without large median tooth; pedipalpal femur and patella smooth (sometimes very rare and weak granules on outer side of femur); chelal hand with faint reticulate ornament; notch on median side of patella reaching almost middle of patellar club length; movable finger distinctly longer than hand^+^ but almost equal to femur in length; trichobothrium *ist* situated distal to middle of fixed chelal finger; distal half of fixed chelal finger with teeth almost equal in size and shape; anterolateral process of coxa of leg I pointed and slightly enlarged, its mediolateral process slightly prominent, denticulate; sternite II with 5 setae; pedipalpal femur 4.5–5.1× as long as broad (0.77–1.00/0.15–0.22), chelal hand^+^ 1.62 × as long as broad (0.62–0.71/0.38–40); movable chelal finger length 0.79–0.95.

###### Description.

♂ and ♀. Coloration: carapace distinctly darker than tergite I and slightly paler than pedipalp; pedipalpal segments uniformly coloured.

**Figure 4.**
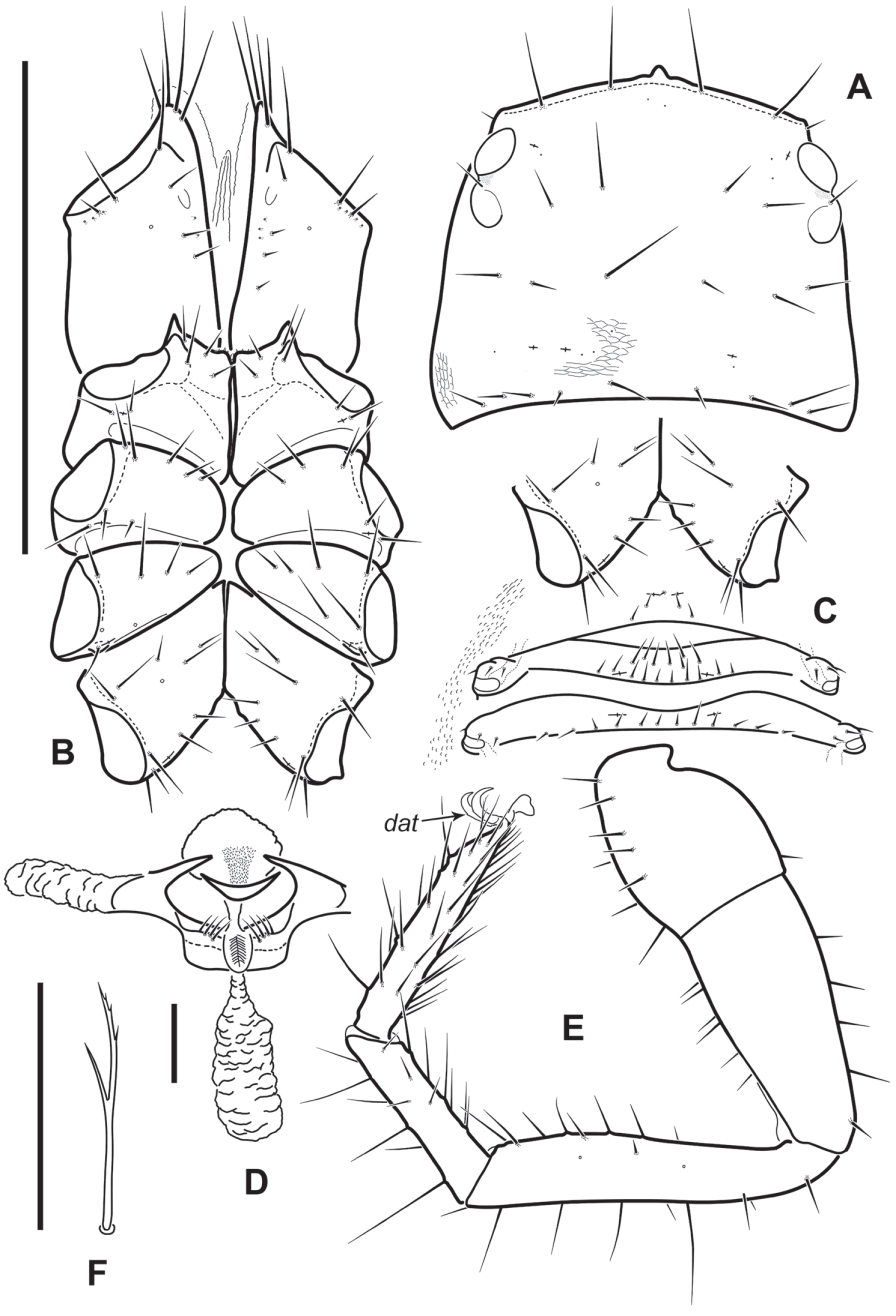
Neobisium (Neobisium) adjaricum sp. nov., holotype male **A** carapace, dorsal view **B** coxae of pedipalp and legs I–IV, ventral view **C** sternites II–IV, ventral view **D** male genitalia, ventral view **E** right leg IV (without trochanter), dorsal view **F** sub-terminal seta on pedal tarsus IV. Abbreviation: *dat* – dorsal accessory tooth. Scale bars: 1.0 mm (**A–C, E**); 0.1 mm (**D, F**).

Carapace (Figs 4A, 6C, H, J): with posterior border partially unsclerotised and pale, covered with fine reticulate ornament (Fig. 6H), wider than long, 0.79 × (0.83–0.9 ×) as long as broad, with two pairs of corneate eyes, anterior eyes slightly larger than posterior ones; distance between anterior margin of anterior eye and anterior margin of carapace 0.05, diameter of anterior eye 0.10 (0.08–0.09), diameter of posterior eye 0.09 (0.07–0.09), distance between eyes 0.02; carapace with 27 (23–24) setae, its anterior margin with 6 (4–6) setae: 4 macrosetae and 2 marginal microsetae (one paratype without these microsetae); posterior margin with 9 (6–7) setae, chaetotaxy: m4m:6:6:9 (m4m:6:6:6, 4:6:6:7); epistome prominent, short and apically rounded, 0.025/0.040; glandular pores present; anterolateral corners with two small protuberances located below surface of carapace; carapace with 6 (5–6) microlyrifissures: one pair situated in ocular zone, close to anterior eyes, and two pairs located at posterior margin.

Tergites weakly sclerotised; all setae simple; tergite X with 2 (2–4) pseudotactile and 2 tactile setae; tergite XI with 4 tactile setae; chaetotaxy: 6:9:11:11:11:12:12:13:11:4T2T4:T2T2T2T:2 (6:8–9:10–11:11–12:11:12:11:10–12:11:4T1–2T4:T2T2T2T:2).

Sternites entirely smooth, weakly sclerotised; anterior operculum of males with 5 and posterior operculum of males with 17 (16–17) setae, of which 7 (6–7) setae located close to genital aperture (Fig. 4C); sternite II of female with 6 setae, sternite III of female: (3)18(3). All setae simple; those on sternites IV–XI uniseriate; chaetotaxy: 5:(3)17(3):(3)12(3):16:16:16:16:16:6T1T6:T2T3T2T:2 (5–6:(3)16–18(3):(3)12–13(3):15–17:14–15:15:14–16:13–15:4–6T1T4–6:T3T2T3T:2).

Internal genitalia with moderately long lateral and median genital sacs, median sac wrinkled, same length as lateral sacs; genital opening with 4+4 internal setae (Fig. 4D).

**Figure 5.**
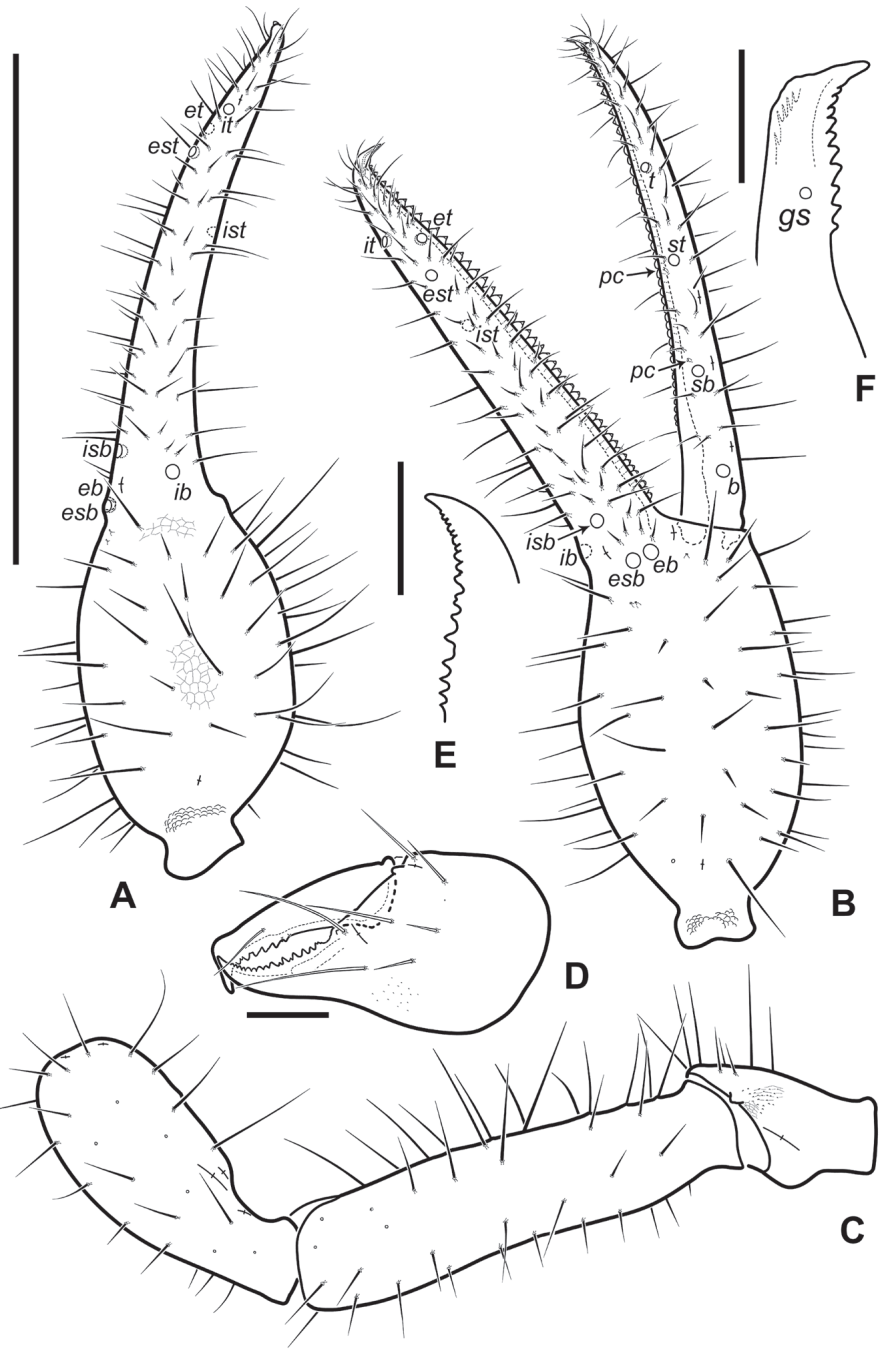
Neobisium (Neobisium) adjaricum sp. nov., holotype male (**A–D**) and paratype male (**E, F**) **A** left chela, dorsal view **B** right chela, lateral view **C** right pedipalp (without chela), dorsal view **D** left chelicera, dorsal view **E** fixed finger of left chelicera, partial dorsal view **F** movable finger of left chelicera, partial dorsal view. Abbreviations: trichobothria on fixed chelal finger: *eb* – exterior basal, *esb* – exterior sub-basal, *est* – exterior sub-terminal, *et* – exterior terminal, *ib* – interior basal, *isb* – interior sub-basal, *ist* – interior sub-terminal, *it* – interior terminal; trichobothria on movable chelal finger: *b* – basal, *sb* – sub-basal, *st* – sub-terminal, *t* – terminal; *gs* – galeal seta; *pc* – coupled sensilla. Scale bars: 1.0 mm (**A–C**); 0.1 mm (**D–F**).

**Figure 6.**
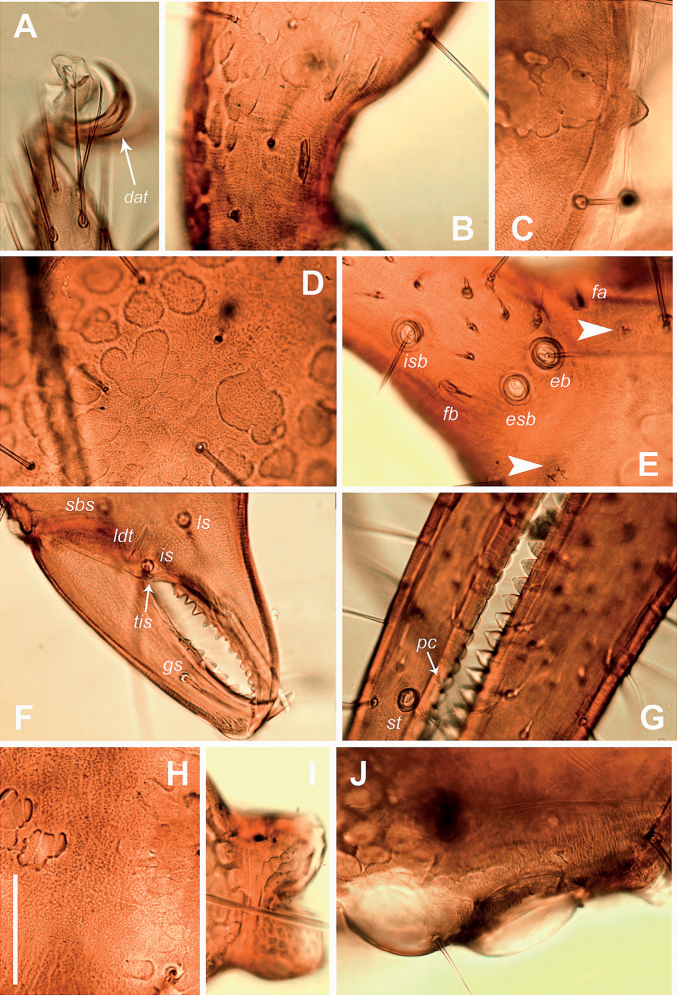
Neobisium (Neobisium) adjaricum sp. nov., holotype male, light microscope images **A** distal part of left pedal tarsus IV, dorsal view **B** basal part of pedipalpal patella, dorsal view **C** epistome, dorsal view **D** surface of chelal hand, dorsal view **E** basal part of right chelal fixed finger, lateral view **F** part of left chelicera (part), dorsal view **G** part of chelal fingers, lateral view **H** surface of carapace, dorsal view **I** pedicel of chela, lateral view **J** right eyes, dorsal view. Abbreviations: trichobothria on fixed chelal finger: *eb* – exterior basal, *esb* – exterior sub-basal, *isb* – interior sub-basal; trichobothria on movable chelal finger: *st* – sub-terminal; *dat* – dorsal accessory tooth; *fa*, *fb* – lyrifissures; *gs*, *ls*, *is*, *sbs* – cheliceral setae; *pc* – coupled sensilla; *tis* – tooth close to seta *is* on chelicera; arrows show glandular pores. Scale bar: 0.1 mm.

Pleural membrane granulated. Chelicera (Figs 5D–F, 6F): hand with 7 acuminate setae; galea knob-like, with poorly developed hyaline convexity, sub-galeal seta situated distal to middle (T = 0.7); hand covered with faint reticulate pattern with pointed corners (Fig. 5D); small tooth (*tis*) under interior seta (*is*) present, triangular (Fig. 6F); fixed finger with 18 (17–19) teeth reaching finger base; movable finger with 7 (8–10) teeth reaching distal to middle of segment, large median tooth absent; serrula interior with 22 (22–25) blades, serrula exterior with 26 (25–28) blades; rallum with 8 blades, two distal ones denticulate, 6 posterior blades simple, smooth and acuminate, 2 or 3 proximal blades smallest.

Coxae (Fig. 4B): pedipalpal coxa excluding manducatory process with 7 or 8 setae, manducatory process with 4 acuminate setae, seta at base of manducatory process longest; coxa I with moderately long triangular, sclerotised, apically pointed anterolateral process (0.06/0.04), and with denticulate mediolateral process; coxal chaetotaxy of legs: 6:6–8:7:10–11 (5–6:6–8:7:10–11). Each coxa of legs with one lyrifissure, pedipalpal coxa with one maxillary lyrifissure.

Pedipalp (Figs 5A–C, 6B, D, E, G, I): femur and patella smooth (in one paratype, outer side of femur finely granulate), hand of chela covered with fine reticulate ornament (Fig. 6D), pedicel of chela with distinct ornament; trochanter with small dorsal tubercle, 2.0 × (1.60–1.85 ×) as long as broad; femur with short pedicel, its margins of femur without tubercles, some setae mostly located in basal half of segment, without enlarged alveoli, one glandular pore located dorsodistally, 4.5 × (5–5.1 ×) as long as broad; patella with short and stout pedicel (0.20–0.22), patella distinctly shorter than femur, 1.43 × (1.54–1.66 ×) as long as broad, with 3 lyrifissures basally and 2 lyrifissures distally, notch on median side reaching very close to middle of club length. Chela^+^ 3.8 × (3.68–3.87 ×) and chela- 3.7 × (3.52–3.75 ×) as long as broad; movable finger distinctly longer than hand^+^, 1.31 × (1.27–1.33 ×), but almost equal to femur in length, 0.95 × (0.95–1.02 ×); hand^+^ 1.62 × (1.63–1.77 ×) and hand- 1.52 × (1.57–1.62 ×) as long as broad; retrolateral surface of hand with 3 glandular pores located around trichobothria *eb* and *esb* (two pores close to each other, one distant); fixed finger with 3 lyrifissures: *fa* located close to base in retrolateral view, *fb* located slightly proximal to *ib*, *fd* located distal to *it*; movable finger with 3 lyrifissures in retrolateral view: *ma*_2_ located closer to trichobothrium *b* than to *sb*, *ma*_1_ nearly at same level with *sb* (slightly proximal to *sb*), *ma*_3_ closer to *st* than to *sb*, *ma*_1_ slightly closer to *ma*_3_ than to *ma*_2_; one or two sensilla *pc* located between trichobothria *sb* and *st*, close to dental canals (one sensillum closer to *sb*, other closer to *st*; latter not present in all specimens); fixed finger with 48 (48–51) contiguous triangular teeth reaching level of trichobothrium *ib*, two outer distal teeth smallest, other teeth almost equal in size, all teeth with dental canal; movable finger with 45 (44–47) contiguous teeth not reaching level of trichobothrium *b*, teeth slightly reduced in size from tip of base of, distal 10–12 teeth triangular, other blunt and apically rounded, teeth with dental canal (except basal 6 or 7 small teeth); nodus ramosus of venom duct in fixed chelal finger short, situated very close to finger tip.

Trichobothriotaxy: fixed finger with 8 trichobothria, movable finger with 4 trichobothria; fixed finger with trichobothria *esb* and *eb* located close to each other and slightly proximal to *ib*, with *isb* on retrolateral surface, with *ib* closer to *isb* than to *esb* (in lateral and dorsal views), trichobothrium *ist* distinctly closer to *est* than to *isb*, distance *ist*–*est* similar to distance *est*–*it*, but less than twice as long as distance *est*–*et* in lateral view, trichobothrium *ist* situated distal to middle of finger (T = 0.57–0.60), *ist* located distinctly proximal to *t*, *et* located posterior to *it*, at approximately the same distance between *est* and *it* (in lateral and dorsal views), *ist* situated distal to middle of finger; movable finger with trichobothrium *st* situated slightly closer to *t* than to *sb*, trichobothrium *sb* slightly closer to *b* than to *st* (at almost equal distances), distance *b*–*sb* almost equal to distance *st*–*t*.

Legs (Figs 4E, F, 6A): all claws of legs with small dorsal accessory tooth (Figs 4E, 6A), arolia simple and shorter than claws. Leg I femur 4.00 × (4.44–5.50 ×) and patella 3.30 × (3.30–3.40 ×) as long as deep, femur 1.21 × (1.48–1.66 ×) as long as patella, tibia 4.12 × (4.28–4.37 ×), metatarsus 3.00 × (3.12–3.14 ×) and tarsus 4.28 × (4.0–4.28 ×) as long as deep, tarsus 1.25 × (1.20–1.27 ×) as long as metatarsus; leg IV femur 2.00 × (1.76–2.00 ×), patella 2.22 × (2.05–2.63 ×), femur+patella 4.22 × (3.94–4.50 ×), tibia 6.0 × (5.80–6.36 ×), metatarsus 3.75 × (3.75–4.12 ×) and tarsus 5.71 × (5.42–5.75 ×) as long as deep, tarsus 1.33 × (1.26–1.39 ×) as long as metatarsus; tibia IV with long tactile seta situated slightly proximal to middle (T = 0.40), metatarsus IV with long tactile seta situated basally (T = 0.125), tarsus with tactile seta situated proximal to middle (T = 0.38); sub-terminal setae branched, basal ramus short and smooth (Fig. 4F).

Sexual dimorphism not pronounced. Measurements: body length 2.95 (2.87–2.90); carapace 0.65/0.82 (0.64–0.65/0.72–0.77); chelicera 0.45/0.24 (0.40–0.50/0.22–0.26), movable finger of chelicera 0.28 (0.26–0.30); pedipalp: trochanter 0.40/0.20 (0.37–0.40/0.20–0.25), femur 0.90/0.20 (0.77–1.00/0.15–0.20), patella 0.63/0.24 (0.50–0.60/0.20–0.23), chela^+^ 1.52 (1.40–1.55), chela- 1.48 (1.34–1.50), hand^+^ 0.65/0.40 (0.62–0.71/0.38–0.40), hand- 0.61 (0.60–0.65), movable finger 0.86 (0.79–0.95); leg I: trochanter 0.18/0.15 (0.18–0.22/0.15), femur 0.40/0.10 (0.40–0.55/0.09–0.10), patella 0.33/0.10 (0.27–0.33/0.08–0.10), tibia 0.33/0.08 (0.30–0.35/0.07–0.08), metatarsus 0.24/0.08 (0.22–0.25/0.07–0.08), tarsus 0.30/0.07 (0.28–0.30/0.07); leg IV: trochanter 0.40/0.15 (0.40/0.18), femur 0.36/0.18 (0.30–0.40/0.17–0.20), patella 0.40/0.18 (0.37–0.50/0.18–0.19), femur+patella 0.76 (0.67–0.90), tibia 0.60/0.10 (0.58–0.70/0.10–0.11), metatarsus 0.30/0.08 (0.30–0.33/0.08), tarsus 0.40/0.07 (0.38–0.46/0.07–0.08).

###### Comparison.

The new species is most similar to N. (N.) vilcekii and N. (N.) speleophilum to the following characters: tarsal claw IV with dorsal accessory tooth, palpal femur without granulate, trichobothrium *ist* located distinctly proximal to *t.* The new species differs from N. (N.) vilcekii in the following characters: shorter pedipalpal femur (0.77–1.00 vs. 1.35–1.72 in N. (N.) vilcekii), shorter hand^+^ (0.62–0.71 vs. 1.07–1.45) and movable finger (0.79–0.95 vs. 1.32–1.57), higher ratio of length of movable finger to hand^+^ (1.27–1.33 × vs. 1.06–1.23 ×), lower ratio of length to width of chelal hand^+^ (1.62–1.77 × vs. 1.81–2.15 ×), longer epistome (moderately long vs. short), smaller number of setae on sternite II (5 vs. 10–15), longer anterolateral process on coxa I (0.06/0.04 vs. 0.04/0.05), smaller number of setae on pedipalpal and leg coxae (see Table 1), smaller number of setae on manducatory process of pedipalp (4 vs. 5), and the presence/absence of large median tooth on movable finger of chelicera (absent vs. present) (Krumpál 1983; Nassirkhani and Snegovaya 2021). The new species differs from N. (N.) speleophilum in the following characters: shorter chela^+^ (1.40–1.55vs. 1.72–1.80 in N. (N.) speleophilum), hand^+^ (0.62–0.71 vs. 0.73–0.80), and movable finger (0.79–0.95 vs. 1.12–1.20), lower ratio movable finger/hand^+^ (1.27–1.33 × vs. 1.45–1.53 ×), sternite II with 5 setae vs. 11 or 12, longer (as long as lateral sacs) median sac of male genitalia vs. short (lateral sac longer than median) (Krumpál 1986; Nassirkhani et al. 2020).

**Table 1.** Numbers of setae on the coxae of some species of the subgenus Neobisium from the Caucasus and adjacent territories.

Species	Pedipalpal coxa (including manducatory process)	Manducatory process of pedipalpal coxae	Coxa I	Coxa II	Coxa III	Coxa IV
N. (N.) adjaricum sp. nov.	11–12	4	5–6	6–8	7	10–11
N. (N.) anatolicum	9	4	10	7	6	11
N. (N.) artaxerxesi	10–13	5	5–7	5–6	4–6	9–13
N. (N.) catherineae	12	4	6–7	6	5–6	7–8
N. (N.) crassifemoratum	8–9	3	7–9	7–8	5–6	9–11
N. (N.) golovatchi	5–7	4	4–7	4–5	3–5	9–10
N. (N.) kamenskyi	9	5	6	6	6	8
N. (N.) kobachidzei	10–12	4	6–7	7	7	8–13
N. (N.) kovalevskayae	13	5	6–9	6	5–7	6–8
N. (N.) speleophilum	10–12	4	6–7	4–5	4–6	8–10
N. (N.) vilcekii	12–15	5	6–11	7–12	7–11	13–22

###### Distribution.

Known only from two adjacent *Sphagnum* bogs, Ispani 1 and Ispani 2, situated near the town of Kobuleti in the Republic of Adjara (Georgia).

###### Habitats.

In Ispani 1 bog, N. (N.) adjaricum sp. nov. was collected in its northwestern part (site 1), with large relatively dry hummocks (20–40 cm high, 1–2 m wide) and moist flat spaces between them (Fig. 2A). The new species was collected only from hummocks. The hummocks consisted mainly of loose *Sphagnum* (with predominant *S.papillosum*, *S.austinii*, and *S.palustre*; in particular, the latter species was predominant in sample I-37, where one paratype was collected; Fig. 2B), with common *Juncuseffusus* L., *Moliniacaeruleaarundinacea* (Schrank) K. Richt., *Rhododendron* spp., and *Pteridiumtauricum* V.I. Krecz. *Sphagnum* was dry to slightly wet, with pH 2.9–3.0 and mineralization of 0.04–0.08 ppt.

In Ispani 2 bog, N. (N.) adjaricum sp. nov. was collected from a flat site in its (site 3; Fig. 1A) covered by a flat carpet of *Sphagnum*, with *S.papillosum* and *S.palustre* predominant, and with the thinned stands of *M.caeruleaarundinacea* (Fig. 1B). *Sphagnum* was wet to moist, with a pH of 4.0 and mineralization of 0.07 ppt.

The new species seems to be rare, considering that only 1–3 specimens were collected at each site, in 10 quantitative samples (sample size of 0.05 m^2^). As distinct from other pseudoscorpion species, all specimens of N. (N.) adjaricum sp. nov. were collected only from quantitative samples and no specimens were collected by other techniques (sifting, pitfall trapping, and/or rearing from substrata in the lab).

###### Etymology.

The Latin adjective *adjaricum* indicates that the new species is named after the Republic of Adjara (= Ajaria), Georgia, where it was discovered.

##### ﻿ Neobisium (Neobisium) vilcekii

Taxon classificationAnimaliaPseudoscorpionesNeobisiidae

Krumpál, 1983

31DCF749-63A8-501C-AAFA-471D2D4137AE

Figs 3B , 7 , 8 , 9

###### Material examined.

***Holotype*** ♂ (SNM 28SR), **Russia**, North Ossetia–Alania, Prigorodnyi Distr., near Karmadon, 1.VI.1976, Mikuláš J. Lisický leg.

###### Diagnosis.

Carapace with short and apically rounded epistome; two pair of eyes present, lenses of posterior eyes with low convexity; movable cheliceral finger with large median tooth; pedipalpal segments smooth, covered with fine reticulate ornament; notch on median side of pedipalpal patella not extending from distal third of patellar club length; movable finger longer than hand^+^, but almost equal to femur in length; trichobothrium *ist* situated distal to middle of fixed chelal finger; distal half of fixed chelal finger with teeth almost equal in size and shape; anterolateral process of coxa of leg I pointed and relatively short, with mediolateral process slightly prominent, denticulate; sternite II with 15 setae; pedipalpal femur 4.41 × as long as broad (1.41/0.32), chelal hand^+^ 1.89 × as long as broad (1.19/0.63); movable chelal finger length 1.40.

###### Redescription.

♂. Coloration: carapace reddish brown, opisthosoma and legs paler.

**Figure 7.**
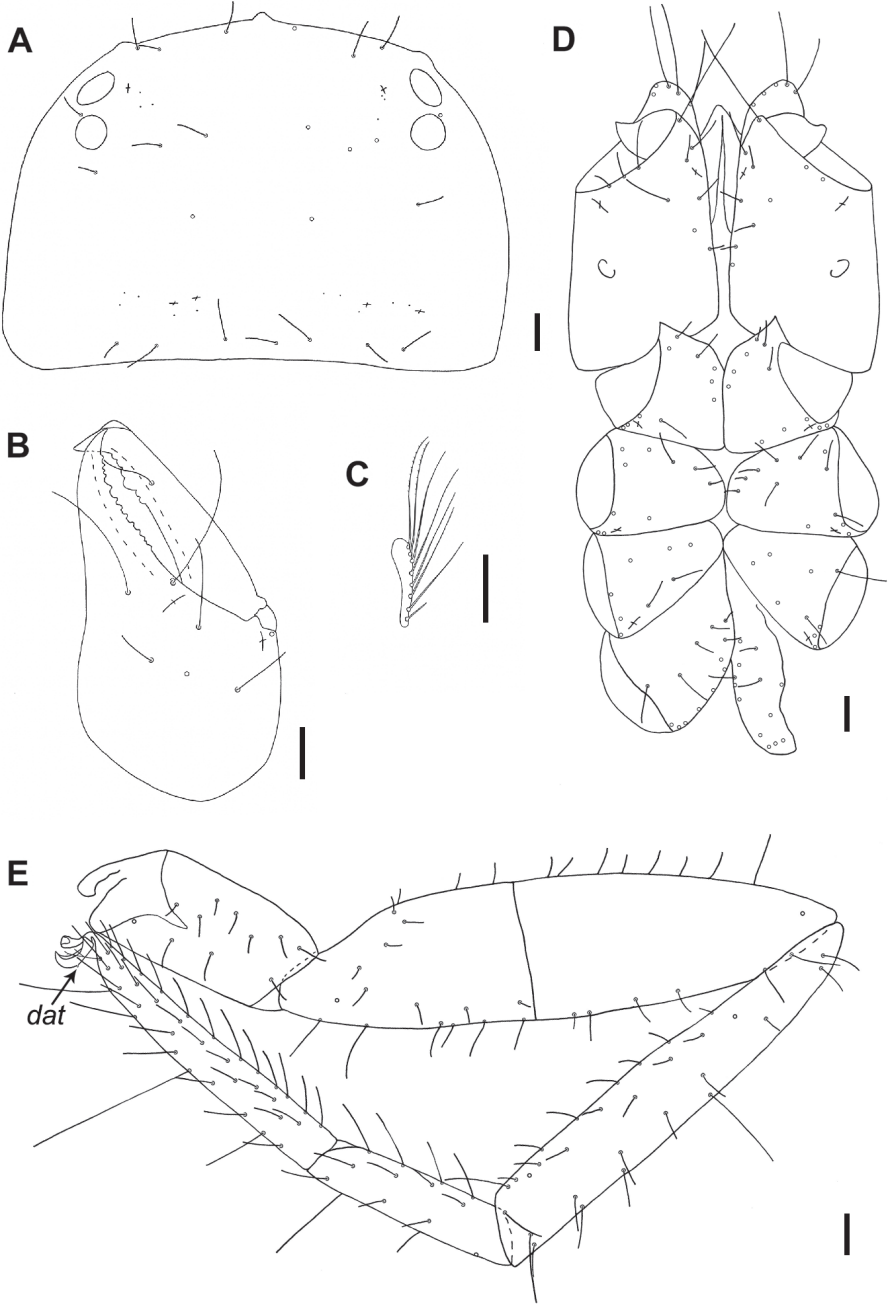
Neobisium (Neobisium) vilcekii Krumpál, 1983, holotype male **A** carapace, dorsal view **B** right chelicera, dorsal view **C** rallum, ventral view **D** coxae of pedipalp and legs I–IV, ventral view **E** left leg IV, dorsal view. Abbreviation: *dat* – dorsal accessory tooth. Scale bars: 0.1 mm.

Carapace (Figs 7A, 9A–C): without transverse furrows, covered with reticulate ornament (Fig. 9A); 0.71 × as long as broad, with two pairs of corneate eyes (Figs 7A, 9B), anterior eyes slightly larger than posterior ones (0.11 vs. 0.09); distance between anterior margin of anterior eye and anterior margin of carapace 0.09, diameter of anterior eye 0.07, diameter of posterior eye 0.08, distance between eyes 0.02; carapace with 24 setae, anterior margin with 6 macrosetae, posterior margin with 7 setae; chaetotaxy: 6:7:4:7; all anterior setae almost equal in length (Fig. 7A); epistome short and apically rounded, 0.03/0.05 (Figs 7A, 9C); glandular pores present (Fig. 7A); anterolateral corners with two small protuberances; carapace with 6 microlyrifissures: one pair situated in ocular zone, close to anterior eyes, and two pairs located at posterior margin (Fig. 7A).

Tergites undivided, posterior ones damaged; all setae acuminate; chaetotaxy of tergites I–IX: 5:6:11:12:11:12:12:13:12.

**Figure 8.**
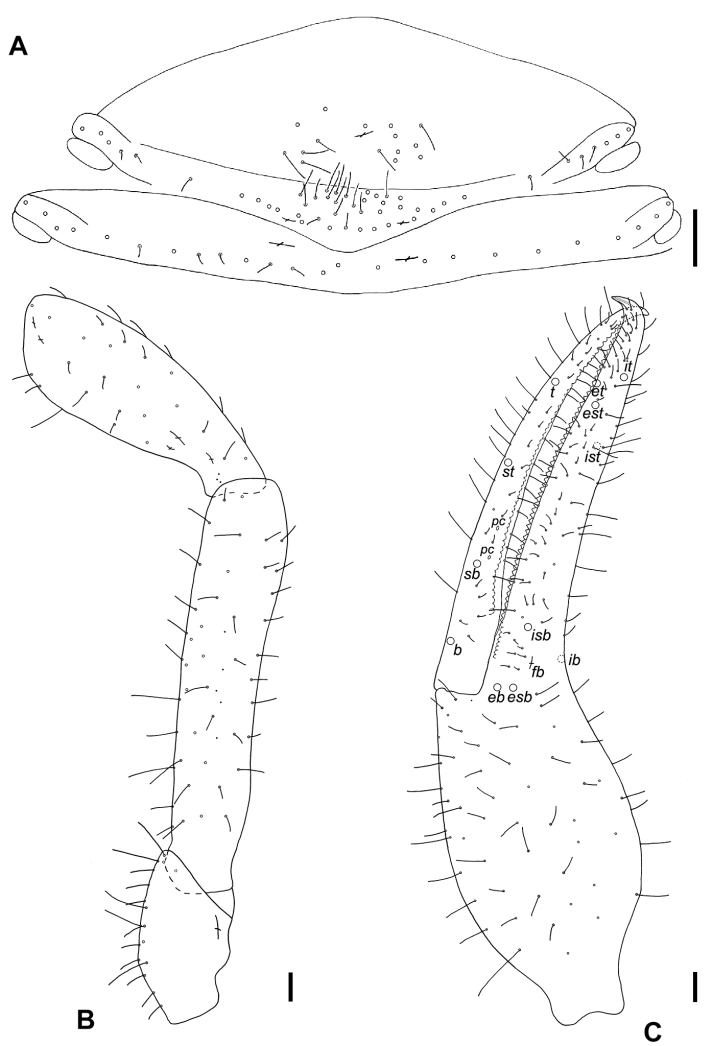
Neobisium (Neobisium) vilcekii Krumpál, 1983, holotype male **A** sternites II–III, ventral view **B** left pedipalp (without chela), dorsal view **C** left chela, lateral view. Abbreviations: trichobothria on fixed chelal finger: *eb* – exterior basal, *esb* – exterior sub-basal, *est* – exterior sub-terminal, *et* – exterior terminal, *ib* – interior basal, *isb* – interior sub-basal, *ist* – interior sub-terminal, *it* – interior terminal; trichobothria on movable chelal finger: *b* – basal, *sb* – sub-basal, *st* – sub-terminal, *t* – terminal; *fb* – lyrifissure, *pc* – coupled sensilla. Scale bars: 0.1 mm.

Sternites undivided, posterior ones damaged; all setae acuminate; anterior operculum with 15 setae and one lyrifissure, posterior operculum with 38 setae, of which 19 located close to genital aperture and two lyrifissures (Fig. 8A); chaetotaxy of sternites II–IX: 15:(5)38(6):(4)16(4):19:17:15:15:15.

**Figure 9.**
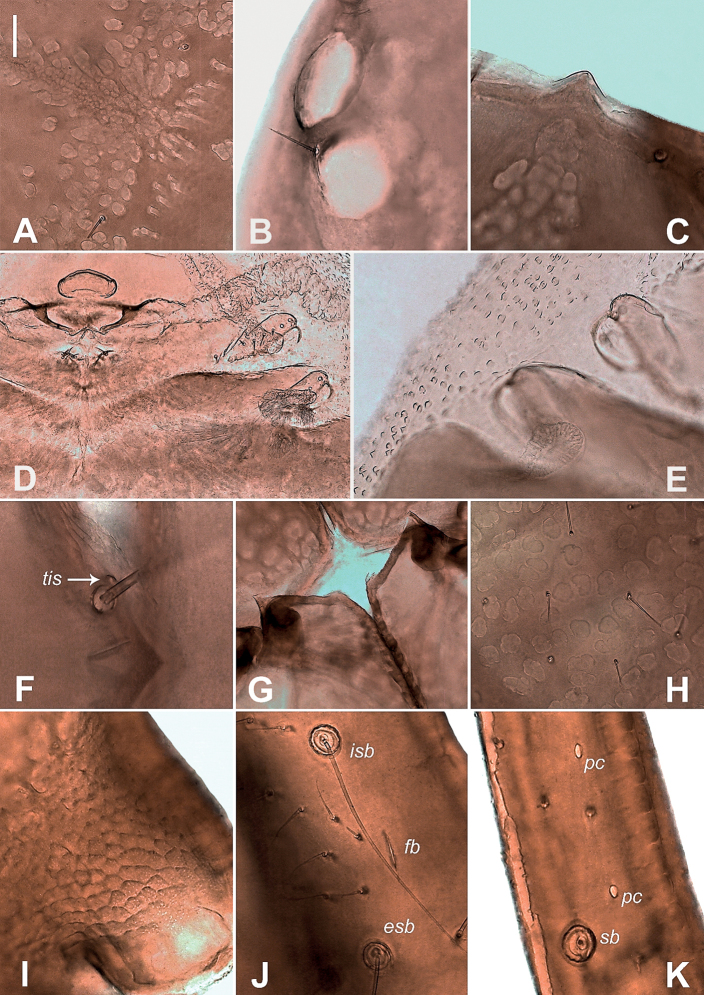
Neobisium (Neobisium) vilcekii Krumpál, 1983, holotype male, light microscope images **A** detail of ornamentation on carapace, dorsal view **B** left eyes, dorsal view **C** epistome, dorsal view **D** genitalia, ventral view **E** detail of pleural membrane, dorsal view **F** tooth close to seta *it* on chelicera, dorsal view **G** anterolateral processes on coxae of leg I, ventral view **H** detail of ornamentation on pedipalpal hand, dorsal view **I** detail of pattern on pedicel of pedipalpal hand, dorsal view **J** lyrifissure on fixed chelal finger, lateral view **K** coupled sensilla on movable fixed finger, lateral view. Abbreviations: trichobothria on fixed chelal finger: *esb* – exterior sub-basal, *isb* – interior sub-basal; trichobothria on movable chelal finger: *sb* – sub-basal; *fb* – lyrifissure, *pc* – coupled sensilla; *tis* – tooth close to seta *is* on chelicera. Scale bar: 0.1 mm.

Internal genitalia (Fig. 9D) with moderately long lateral and median genital sacks, median sack wrinkled; genital opening with 8+7 internal setae.

Pleural membrane granulated (Fig. 9E). Chelicera (Figs 7B, C, 9F): 1.51 × as long as broad; hand with 7 acuminate setae and two lyrifissures (Fig. 7B); galea knob-like, with poorly developed hyaline convexity; sub-galeal seta situated distal to middle (T = 0.11) of movable finger; small tooth (*tis*) under interior seta *is* present, blunt (Fig. 9F); hand covered with faint reticulate pattern with pointed corners; fixed finger with 13 teeth reaching finger base, movable finger with 6 teeth reaching middle of finger, large median tooth present; serrula interior with 20 blades, serrula exterior with 27 blades; rallum (Fig. 7C) with 8 blades, two distal ones denticulate, 6 posterior blades simple, smooth and acuminate, 3 proximal blades smallest.

Coxae (Figs 7D, 9G): pedipalpal coxa excluding manducatory process with 10 setae, manducatory process with 5 acuminate setae; coxa I with short triangular, sclerotised, apically pointed anterolateral process (0.04/0.05) (Fig. 9G), mediolateral process denticulate; coxal chaetotaxy of legs: 10–11:10–12:8–9:13, all setae acuminate; lyrifissures: two on each pedipalpal coxa, one on each coxa I–III of legs, not visible on pedal coxae IV; pedipalpal coxa with one maxillary lyrifissure.

Pedipalp (Figs 8B, C, 9H–K): pedipalpal segments smooth (Fig. 9H); all setae acuminate; trochanter with small dorsal tubercle and one lyrifissure (Fig. 8B), 2.03 × as long as broad; femur with short pedicel, margins of femur without tubercles, 5 glandular pores present (Fig. 8B), 4.41 × as long as broad; patella with short pedicel, 3 glandular pores present (Fig. 8B), 2.67 × as long as broad, with 3 lyrifissures basally and 2 distally (Fig. 8B); chela^+^ 3.76 × and chela- 3.57 × as long as broad; movable finger distinctly longer than hand^+^, 1.18 ×, but almost equal to femur in length, 0.99 ×; hand^+^ 1.89 × and hand- 1.68 × as long as broad, pedicel with faint reticulate pattern with pointed corners (Fig. 9I); retrolateral surface of hand with one glandular pore located near trichobothrium *eb* (Fig. 8C); fixed finger with one lyrifissure: *fb* located slightly proximal to trichobothrium *ib* (Fig. 9J), *fa* and *fd* not visible; lyrifissures on movable finger not visible; two coupled sensilla *pc* situated between *st* and *sb* (Figs 8C, 9K); fixed finger with 65 contiguous triangular teeth reaching level of trichobothrium *ib*, teeth almost equal in size; movable finger with 54 contiguous teeth not reaching level of trichobothrium *b*, teeth blunt and apically rounded; nodus ramosus of venom duct in fixed chelal finger short, situated very close to finger tip (Fig. 8C).

Trichobothriotaxy (Fig. 8C): fixed finger with 8 trichobothria, movable finger with 4 ones; fixed finger with close-set trichobothria *esb* and *eb* located slightly proximal to *ib*, with *isb* on retrolateral surface, *ib* closer to *isb* than to *esb*, *ist* distinctly closer to *est* than to *isb*, distance *ist*–*est* approximately the same as distance *est*–*it*, but less than twice as long as distance *est*–*et* in lateral view, trichobothrium *ist* situated distal to middle of finger, *ist* located distinctly proximal to *t*, *et* located proximal to *it*, at approximately the same distance between *est* and *it* (in lateral and dorsal views), *ist* situated distal to middle of finger; movable finger with trichobothrium *st* situated slightly closer to *t* than to *sb*, trichobothrium *sb* slightly closer to *b* than to *st*, distance *b*–*sb* almost equal to distance *st*–*t*.

Legs (Fig. 7E): all claws of legs with small dorsal accessory tooth, arolia simple and shorter than claws. Leg I trochanter 1.42 ×, femur 5.79 ×, patella 4.07 ×, tibia 5.82 ×, metatarsus 3.80 × and tarsus 5.40 × as long as deep. Leg IV trochanter 2.11 ×, femoropatella 3.75 ×, tibia 6.33 ×, metatarsus 4.23 × and tarsus 6.82 × as long as deep; tibia IV with long tactile seta situated slightly proximal to middle (T = 0.21 in length, 0.52 from base), metatarsus IV with long tactile seta situated basally (visible only base of trichobothrium, 0.08 from base), tarsus IV with tactile seta situated approximately in middle of segment (T = 0.35, 0.37 from base); sub-terminal setae branched.

Measurements: body length 3.65; carapace 0.92/1.30; chelicera 0.59/0.39, movable finger of chelicera 0.42; pedipalp: trochanter 0.69/0.34, femur 1.41/0.32, patella 1.04/0.39, chela^+^ 2.37, chela- 2.25, hand^+^ 1.19/0.63, hand- 1.06, movable finger 1.40; leg I: trochanter 0.34/0.24, femur 0.81/0.14, patella 0.57/0.14, tibia 0.64/0.11, metatarsus 0.38/0.10, tarsus 0.54/0.10; leg IV: trochanter 0.57/0.27, femur+patella 1.35/0.36, tibia 1.14/0.18, metatarsus 0.55/0.13, tarsus 0.75/0.11.

###### Remarks.

The states of several characters reported here slightly differ from those in the original description of N. (N.) vilcekii (Krumpál 1983), viz. the number of setae on the posterior carapace margin (7 vs. 8); number of setae on the tergites III (11 vs. 10), V (11 vs. 12) and VIII (13 vs. 12); number of setae on the sternites III [(5)38(6) vs. (6)37(6)], VI (17 vs. 16), VIII and IX (each with 15 vs. each with 14); number of internal setae in the genital opening (8 + 7 vs. 7 + 7); number of teeth on the fixed and movable cheliceral fingers (13 and 6 vs. 11 and 4); number of setae on the coxae II of legs (10–12 vs. 11–12) and pedal coxae III (8–9 vs. 9–11) and IV (13 vs. 12–13); body length (3.65 vs. 3.04); carapace (0.92/1.30 vs. 0.69/0.95); pedipalpal trochanter (0.69/0.34 vs. 0.60/0.32). The numbers of setae on two last tergites and sternites are missing in the present redescription because these segments were damaged on the slide mount, but the respective data are given in the original description (tergites X with 12, XI with 10 and sternites X with 16, XI with 12 setae; see Krumpál 1983). Several new characters were added in the present redescription, namely, the measurements and ratio of length to width of chelicera, measurements and ratio of length to depth of trochanters of leg I and leg IV, presence of glandular pores, number of lyrifissures on carapace, number of blades on serrula exterior and interior, presence of tooth *tis* on chelicera, number of lyrifissures on palps and coxae.

The holotype differs from the specimens described by Nassirkhani and Snegovaya (2021) in the following characters: the number of setae on the anterior carapace margin (6 vs. 4), number of setae on the sternites II (15 vs. 10–14) and III (38 vs. 39–47); number of setae around male genital aperture on the sternite III (19 vs. 21–27), number of internal setae in the genital opening (8 + 7 vs. 5–6+5–6), number of teeth on fixed and movable cheliceral fingers (13 and 6 vs. 15–18 and 10–12), chela^+^ (3.76 × vs. 4.00–4.33 ×) and chela- of the male (3.57 × vs. 3.84–4.12 ×); chelal hand^+^ (1.89 × vs. 1.98–2.15 ×) and hand- of the male (1.68 × vs. 1.73–1.89 ×), number of the *pc* sensilla (two, between *st* and *sb*, vs. one, proximal to *sb*), number of teeth on the movable chelal finger of male (54 vs. 56–62), and number of setae on the coxa IV (13 vs. 15–22). The states of most characters mentioned here are within the variability range of this species (Krumpál 1983; Nassirkhani and Snegovaya 2021).

###### Distribution.

The species is known only from two localities in the Republic of North Ossetia–Alania (Russia) in the North Caucasus: the type locality near Karmadon (ca. 1500 m a.s.l.), and another locality in the valley of the Terek River in the environs of Mozdok (115 m a.s.l.) that was reported by Nassirkhani and Snegovaya (2021). The localities are situated at different altitudes, hence they should strongly differ from each other in the landscape and climatic conditions.

###### Habitats.

The habitat of N. (N.) vilcekii near Karmadon is unclear (Krumpál 1983). It is questionable whether the species is confined to mire or wet habitats. The material from the environs of Mozdok that was used by Nassirkhani and Snegovaya (2021) for redescription was collected in a floodplain, in litter consisting of leaves of broad-leaved trees mixed with wood pieces. Hence, it is possible that N. (N.) vilcekii is a hygrophilous species.

## ﻿Discussion

Various characters are used in the taxonomy of species belonging to the subgenus Neobisium (genus *Neobisium*), but the value of some characters is questionable. For example, Nassirkhani et al. (2020) discussed the morphological features of N. (N.) anatolicum from different locations (records of this species from several regions are doubtful and require confirmation) and indicated the high variability of many characters in the specimens from different regions. The diagnostic characters of *Neobisium* s. str. are poor and descriptions of many species are with insufficient data. Hence, it is important to study in detail the characters and intraspecific variability for as many species of the subgenus as possible.

We have ascertained that the males of two closely related species, N. (N.) vilcekii and N. (N.) adjaricum sp. nov., among other things, markedly differ in the number of setae on the sternite II (10–15 vs. 5). The same number of these setae is observed in all males of the type series of N. (N.) adjaricum sp. nov. Furthermore, the relative position of these setae is the same in all three males of the new species. The only female of N. (N.) adjaricum sp. nov. has 6 setae on ist sternite II. Variability number of setae on male sternite II was observed in some other Caucasian species of the subgenus, for example, in N. (N.) catherineae (9–10), N. (N.) speleophilum (11–12), N. (N.) kovalevskayae (8–9), N. (N.) artaxerxesi (8–9), N. (N.) golovatchi (11–14), and N. (N.) crassifemoratum (11–13) (Nassirkhani and Doustaresharaf 2018; Nassirkhani et al. 2018, 2019, 2020), however, the variation within each of the species was 1–3 setae. Maximum variation in this character was observed in N. (N.) vilcekii, namely 5 (10–15 setae) (Nassirkhani and Snegovaya 2021; this study).

The variation reported in the above-mentioned species does not take into account possible differences between males and females. In this regard, it is possible to state that number of setae on male sternite II is stable enough to distinguish species, with some variability being possible. It should be noted that none of the 13 specimens of N. (N.) vilcekii collected from the floodplain of the Terek River had 15 setae on the sternite II (as was the case with the holotype of the same species), but all had between 10 and 14 setae (Nassirkhani and Snegovaya 2021).

In contrast, the number of setae on the carapace (anterior and posterior rows) in the type specimens of N. (N.) adjaricum sp. nov. varies noticeably, i.e., 4 or 6 in the anterior row (microsetae present or absent), 6, 7, or 9 in the posterior row. Variability in the number of setae in the posterior row was previously observed in some species in the subgenus Neobisium, i.e., N. (N.) carcinoides (4–10), N. (N.) kovalevskayae (5–6) and N. (N.) artaxerxesi (7–9) (Mahnert 1988; Nassirkhani et al. 2018, 2019), but such variability in the anterior row was recorded here for the first time. Interestingly, none of the 13 specimens of N. (N.) vilcekii from the floodplain of the Terek River contained 6 setae in the anterior row on the carapace (Nassirkhani and Snegovaya 2021). Keeping this in mind one should use the number of setae in the anterior and posterior rows on the carapace for species diagnostics with caution, if other good distinguishing characters are missing.

Examination of the type series of N. (N.) adjaricum sp. nov. also demonstrated relative stability of the shape and position of teeth on the chelicera, primarily the presence or absence of the large median tooth/teeth on the movable finger. In this character, N. (N.) adjaricum sp. nov. clearly differs from N. (N.) vilcekii, which only sometimes possesses a small median tooth on the movable finger (Nassirkhani and Snegovaya 2021).

The position of the sensillum *pc* is rarely mentioned descriptions of species belonging to the subgenus Neobisium. Recently the position of *pc* in relation to trichobothria on the movable chelal finger is considered a character for distinguishing species in some families of pseudoscorpions (e.g., Chthoniidae) (Zaragoza 2017; Turbanov and Kolesnikov 2021). In N. (N.) adjaricum sp. nov., variability in the number of *pc* (one or two) is observed, however, two *pc* were found only in one specimen, with the anterior *pc* indistinct (shifted apart from the level of the teeth line). In the holotype of N. (N.) vilcekii, two *pc* are distinctly visible between *st* and *sb*, whereas Nassirkhani and Snegovaya (2021) reported for this species only one *pc* (as *se*) proximal to *sb*. At the same time, the former authors illustrated an unnamed structure near *st* (Nassirkhani and Snegovaya 2021: fig. 1H), which resembles the second *pc* in N. (N.) adjaricum sp. nov. (Fig. 6G). Additional studies on other related species are necessary to reveal the variability or stability of this character.

We have demonstrated that N. (N.) adjaricum sp. nov. and N. (N.) vilcekii noticeably differ in the number of setae on the coxae of pedipalps and legs, as well as in the number of setae on the pedipalpal manducatory process. Although these numbers partially overlap (i.e., minimum values for one species with maximum ones for another), the upper and lower limits of variation strongly differ. In Table 1, numbers of setae on the coxae of pedipalps and legs are listed for 11 species of *Neobisium* s. str. from the Caucasus and adjacent territories (Nassirkhani and Doustaresharaf 2018; Nassirkhani et al. 2018, 2019, 2020; Nassirkhani and Snegovaya 2021; Nassirkhani 2022). It is worth mentioning that the number of setae on the pedipalpal manducatory process does not vary within species, so it can be used for species diagnostics (this study). It should be noted that Mahnert (1988) points to the variability of this trait in European populations of N. (N.) carcinoides (4–5 or 3–6), while leaving open the question of the possibility of the existence of a cryptic species complex. Some species [e.g., N. (N.) vilcekii] are characterised by an increased number of setae on the coxae of legs, however, this is noticeable only when the number of setae differs considerably between species. To objectively assess this character, it is necessary to examine a large series of specimens from the same population for different species.

An isolated tooth *tis* is present close to the interior seta of the chelicera in N. (N.) adjaricum sp. nov. and the holotype of N. (N.) vilcekii. Other researchers did not mention the presence of this tooth for *Neobisium* s. str., including Nassirkhani and Snegovaya (2021), who redescribed N. (N.) vilcekii. However, it may be hardly visible due to its small size and close position to the seta *is*. We found the same tooth in the genus *Microbisium* Chamberlin, 1930 from the *Sphagnum* bogs of Georgia (this study), but did not observe it in many species of the families Syarinidae Chamberlin, 1930 and Chthoniidae Daday, 1888 (Kolesnikov and Turbanov 2018; Kolesnikov et al. 2019; Turbanov and Kolesnikov 2020, 2021). At present, the question remains whether this tooth is present in all species of Neobisiidae or it exists only in some genera and species. The shape of the tooth in N. (N.) adjaricum sp. nov. differs from that in N. (N.) vilcekii (triangular and rounded, respectively; Figs 6F, 9F).

## Supplementary Material

XML Treatment for Neobisium (Neobisium) adjaricum

XML Treatment for Neobisium (Neobisium) vilcekii
